# Household environmental management practices, community engagement, and self-reported malaria incidence in urban and rural communities of Malawi

**DOI:** 10.3389/fpubh.2026.1919768

**Published:** 2026-10-14

**Authors:** Memory Magdalene Lungu, Arsen Davitadze

**Affiliations:** 1 Institute of International Development and Partnership, ITMO University, St. Petersburg, Russia; 2 International Centre for Health Economics, Management, and Policy, HSE University, St. Petersburg, Russia

**Keywords:** community engagement, environmental management, malaria incidence, Malawi, negative binomial regression, preventive measures

## Abstract

Malaria control in Malawi relies primarily on long-lasting insecticide-treated nets (LLITNs) and indoor residual spraying (IRS), but transmission remains heterogeneous across districts. Previous research indicates that community-based environmental management strategies, such as routine drainage maintenance, vegetation removal, and effective household waste disposal, may reduce environmental conditions conducive to malaria vector proliferation. This study investigated the association between environmental management practices at both household and community levels and self-reported cases of malaria in Malawi. We disseminated an online survey through social media from December 2024 to February 2025, targeting adults in both rural and urban locales, resulting in 282 participants. Negative binomial regression was employed to assess the relationship between preventive measures, household-level environmental management practices, community engagement, and malaria cases during the past year. LLITNs were the most used preventive measure (62.1%). Their use was significantly associated with fewer malaria cases in rural areas (*p* = 0.035) but more malaria cases in urban areas (*p* = 0.018). Household-level (94.3%) and community-level (70.9%) environmental management practices were widely reported but showed weak and area-dependent associations with malaria incidence. Regardless of area of residence, respondents faced difficulties maintaining these practices, mostly due to time constraints (49.3%) and financial constraints (61.3%). Strengthening community education, resource support, and integration of environmental management into existing programs is likely to be needed to sustain these practices. The contrasting rural–urban patterns may reflect unmeasured differences in local transmission intensity and warrant investigation in studies with geographically resolved data.

## 1 Introduction

Malaria is a significant public health challenge, with almost half of the global population vulnerable to infection (1). The infection is induced by the bite of female *Anopheles* mosquitoes that are infected with *Plasmodium* spp. (*P. falciparum*, *P. vivax*, *P. malariae*, *P. ovale*, and *P. knowlesi*) (2). The early clinical expressions of malaria are often nonspecific and resemble influenza-like illness. Fever is the hallmark symptom and, when accompanied by chills and sweating, aids in differentiating malaria from several other tropical febrile illnesses (3).

The World Health Organization’s (WHO) World Malaria Report indicated that there were approximately 263 million malaria cases in 2023, reflecting an increase of 11 million cases compared to the previous year. The WHO African Region accounted for 94% of all cases. In 2023, Malawi recorded 4.81 million confirmed cases, accounting for 1.8% of the global total (4).

The WHO recommends indoor residual spraying (IRS) and long-lasting insecticide-treated nets (LLITNs) as the primary interventions for malaria vector control. LLITNs are bed nets treated with insecticides, most commonly pyrethroids, which reduce mosquito-human contact indoors at night (5). IRS involves the periodic application of residual insecticides to the interior walls of houses, killing mosquitoes that rest on treated surfaces (6). Despite substantial progress in malaria control, these measures are frequently insufficient to interrupt transmission, particularly in high-transmission settings. Outdoor resting and biting behavior (exophily and exophagy) have become more prevalent among vector populations, which limits the effectiveness of interventions designed to target indoor mosquitoes (7). *Anopheles* species in West Africa have also been reported to bite earlier in the evening, further reducing the protection afforded by interventions timed to periods of indoor sleeping (8). Residual transmission persisting despite high coverage of these interventions is now recognised as a major obstacle to further progress (9). A recent case–control study in Benin found that LLITNs and IRS, alone or in combination, did not confer significant protection against malaria, a finding attributed in part to increasing insecticide resistance (5).

Because LLITNs and IRS act on adult mosquitoes at the point of contact with people indoors, neither targets the aquatic stages of the vector. Community-based environmental management (CBEM) has therefore emerged as a complementary approach, reducing the standing water in which *Anopheles* larvae develop and thereby lowering the adult vector population, including mosquitoes that rest and bite outdoors. It has been identified as a policy option alongside LLITNs and IRS in settings where a sufficient proportion of larval habitats can be targeted (10). These practices, such as eliminating standing water and maintaining drainage systems, have been associated with reduced transmission in a range of malaria-endemic settings, including urban and rural areas in sub-Saharan Africa. For instance, a study in Tanzania reported that community-based environmental management interventions involving awareness campaigns and cleanup activities to remove debris and standing water from drains reduced mosquito breeding sites and increased community awareness, with lower malaria infection rates in intervention neighborhoods than in control areas (11). In Ethiopia, where 55% of households routinely cleaned stagnant water and 42% cleared bushes around homes, overall engagement in and awareness of CBEM remained low, with only 26% of households participating in organized campaigns and 28% reporting no community discussions on prevention (12). This highlights the need for multi-sectoral involvement across the environmental, agricultural, and health sectors.

Malaria transmission in Malawi has remained markedly heterogeneous over the past two decades, with district-level prevalence varying widely and some districts persisting as high-transmission areas while others have achieved substantial reductions (13). This variation reflects differences in environmental conditions, including seasonal flooding and land use change that create vector breeding habitats, alongside insecticide resistance and uneven coverage of control interventions across regions. Structural and community-driven measures, including wire mesh screening of windows and locally produced repellents, have been reported to reduce vector densities and malaria cases, particularly when combined with health education that sustains community participation (14). Evidence from randomized evaluation is more equivocal: in Chikwawa District, community-led larval source management and house improvement added to national program interventions produced no further reduction in transmission under high bed net coverage (15). Taken together, these findings indicate that the contribution of environmental management depends on local transmission intensity, the coverage already achieved by existing interventions, and the extent of community participation (16).

There remains a critical gap in research on community-based environmental management practices and their direct impact on malaria outcomes (17). To address this knowledge gap, this study examined the association between household- and community-level environmental management practices and self-reported malaria incidence. We additionally examined associations between established preventive measures, including LLITNs, and self-reported malaria incidence. Lastly, we explored potential key factors influencing the sustainability and scalability of these interventions in Malawi’s diverse epidemiological landscape.

## 2 Materials and methods

A nationwide online cross-sectional study was conducted from December 2024 to February 2025 in Malawian communities to examine the association between household and community environmental management practices and self-reported malaria incidence. As per recent national estimates, approximately 82% of Malawi’s population resides in rural areas (18).

The study population included community members aged 18 years and above living in either rural or urban communities of Malawi. Convenience sampling by means of social media platforms (Facebook groups as well as WhatsApp and Telegram channels and group chats) was used to obtain participants for the study (19).

Data were collected using a structured online questionnaire that requested information on the sociodemographic characteristics of the participants (age, sex, educational level, marital status, household size, and area of residence), knowledge of malaria and prevention, mosquito breeding sites, household-level environmental practices, and community engagement. The questionnaire consisted of 38 close-ended mandatory questions and 5 open-ended items. We first performed a pre-test among 10 survey participants to ensure clarity and proper understanding of questions. Since the changes after the pre-test were minimal, responses from the first 10 participants were included in the final sample.

The survey instrument was administered using Google Forms. Respondents were required to sign in to a Google account so that submissions could be restricted to one response per respondent; however, the collection of email addresses was disabled, so no identifying information was transmitted to or stored by the research team, and all responses remained anonymous. The participants were sufficiently informed about the study and its aims before they could access the online survey form. The study was reviewed and approved by the ITMO University Public Health Sciences Review Board (NKP-73/25–22-11-2024).

All questionnaire items were mandatory, and the online platform was configured to prevent submission of incomplete responses. Manual data cleaning procedures were applied to the open-ended question about the support needed to sustain environmental management practices (responses were grouped into categories). Categories were derived inductively from the responses rather than defined in advance. Twenty responses were excluded because of data quality concerns. All analyses were conducted in Python (version 3.10.16). Data management and descriptive statistics used pandas (version 2.2) and NumPy (version 2.2). Poisson and negative binomial regression models were fitted using statsmodels (version 0.14), with the negative binomial dispersion parameter estimated by maximum likelihood.

Descriptive statistics are presented as frequencies and percentages for categorical variables and as means with standard deviations, medians, and ranges for continuous variables. Before modeling, reported counts of malaria cases in the past year exceeding 10 were replaced by the reported household size, on the grounds that a household cannot experience more episodes in a year than it has members and that such values most plausibly reflect misreporting of the reference period. This rule affected four households, whose reported counts of 11, 12, 12 and 20 were replaced by household sizes of 8, 8, 10 and 11, respectively. Statistical analysis reports counts as given by participants; all regression models use the adjusted counts, for which the mean was 1.68 cases per household. A sensitivity analysis repeating every model on the untruncated counts is provided in Supplementary Table S2.

Negative binomial regression was chosen to examine the relationship between malaria preventive measures, household-level and community-based environmental management practices, and the number of reported malaria cases in the past year. The negative binomial model was selected over the Poisson model due to evidence of over-dispersion in the count data (20). The variance of the analysed outcome (4.18) exceeded its mean (1.68) by a factor of 2.49. Overdispersion was confirmed by a likelihood-ratio test of the dispersion parameter against the null hypothesis of no overdispersion, which rejected the Poisson specification (*χ*^2^ (1) = 136.5, *p* < 0.001; estimated dispersion parameter 0.94, SE 0.15), and by the Cameron–Trivedi regression-based test (*t* = 4.67, *p* < 0.001). The ratio of the Pearson *χ*^2^ statistic to its degrees of freedom under the Poisson model was 2.49. In all models, the dispersion parameter was estimated from the data by maximum likelihood jointly with the regression coefficients, rather than fixed *a priori*. Each predictor was modeled separately with the number of malaria cases as the outcome.

Binary predictors were coded yes = 1 versus no = 0; predictors recording the frequency of a practice were coded ordinally (never = 0, rarely = 1, monthly, weekly, or daily = 2) so that incidence rate ratios express the change in expected malaria cases per one-category increase in reported frequency. Analyses were conducted for the full sample and stratified by area of residence to explore spatial differences in associations. Because the sample was predominantly urban (73.4%), whereas approximately 82% of Malawi’s population is rural, and because area of residence was itself strongly associated with malaria counts (urban versus rural IRR = 0.53, 95% CI 0.39–0.71, *p* < 0.001), all full-sample models were additionally refitted with area of residence as an adjustment variable; these results are reported in Supplementary Table S1. Results are presented as incidence rate ratios with 95% confidence intervals and *p*-values. Associations were considered statistically significant at the 5% level and marginally significant at the 10% level.

## 3 Results

### 3.1 Sociodemographic characteristics of participants

There were 282 participants whose responses were retained for analysis. Among these, 55% were females and 45% were males. Their ages ranged from 18 to 52 (29.3 ± 6.5) years. Practically all (92.6%) participants had higher education. Most respondents were single (61%) and lived in urban communities (73.4%). 94% of all participants lived in their communities for over a year (Table 1).

**Table 1:** Sociodemographic characteristics of participants (*n* = 282).

Variable	Category	*n* (%)
Gender	Female	155 (55)
	Male	127 (45)
Education level	Higher education	261 (92.6)
	Secondary education	21 (7.4)
Employment status	Employed	192 (68.1)
	Unemployed	90 (31.9)
Marital status	Single	172 (61)
	Married	104 (36.9)
	Divorced	6 (2.1)
Current residence	Rural	75 (26.6)
	Urban	207 (73.4)
Time living in current community	Less than 1 year	17 (6)
	1–5 years	128 (45.4)
	More than 5 years	137 (48.6)
Variables (continuous)		Mean (SD); Median; Range
Age		29.32 (6.52), 28, 18–52
Number of people living in the household		4.59 (2.29), 4, 1–16

Mean, Standard deviation (SD), Median, Range are reported for variables Age and Number of people living in the household. Other variables report only their absolute and relative values (*n* and %).

### 3.2 Malaria history and prevention practices

Most participants (62.1%) reported a history of malaria in their household during the past year. A total of 491 malaria cases were reported across all participants, with a mean of 1.74 (SD 2.37) cases per household and a median of 1 (range 0–20) (Table 2).

**Table 2:** Malaria history, use of malaria preventive measures and community-based environmental management practices among respondents (*n* = 282).

Variable	Category	*n* (%) unless otherwise stated
Malaria history	No	107 (37.9%)
	Yes	175 (62.1%)
Malaria cases	Number of cases	491
	Mean (SD)	1.74 (2.37)
	Median	1
	Range	0–20
Use of malaria preventive measures	Yes	269 (95.4%)
	No	13 (4.6%)
Common malaria preventive measures	LLITNs	175 (62.1%)
	Insecticide sprays	66 (23.4%)
	Mosquito coils	76 (27%)
	House modifications	17 (6.1%)
Use of household-level environmental management practices	Yes	266 (94.3%)
	No	16 (5.7%)
Common household-level environmental management practices	Sweeping the yard	209 (74.1%)
	Eliminating standing water	132 (46.8%)
	Covering water containers	138 (48.9%)
	Maintenance of drainage systems	112 (39.7%)
	Managing trash cans	126 (44.7%)
	Mowing grass	174 (61.7%)
	Other environmental practices	72 (25.5%)
Engagement in community-level environmental management practices	Yes	200 (70.9%)
	No	82 (29.1%)
Common community-level environmental management practices	Removing stagnant water in open places (markets, hospitals)	169 (84.5%)
	Mowing grass in open places	174 (87.0%)
	Filling potholes	157 (78.5%)
	Sweeping around markets	172 (86.0%)
	Information sharing	151 (75.5%)

Almost all respondents (95.4%) reported using at least one malaria preventive measure. Among the preventive strategies adopted, the use of LLITNs was the most common (62.1%), followed by mosquito coils (27.0%) and insecticide sprays (23.4%). House modifications, such as installing screens or sealing openings, were the least reported method (6.1%) (Table 2).

Most respondents (94.3%) reported actively engaging in household-level environmental management practices. Among them, the most common activities included sweeping the yard (74.1%), mowing grass (61.7%), covering water containers (48.9%), eliminating standing water (46.8%), and managing trash cans (44.7%) (Table 2).

Community-level environmental management practices were reported by 70.9% of respondents. Among those engaged, participation in individual practices ranged from 75.5% for information sharing to 87.0% for mowing grass in open places (Table 2).

### 3.3 Association between malaria incidence, malaria preventive measures, and community-based environmental management practices

The overall use of malaria preventive measures was not associated with malaria incidence in the full sample. In the stratified analysis, use of any preventive measure was associated with fewer malaria cases in rural areas (IRR 0.40, 95% CI 0.17–0.92, *p* = 0.030) but not in urban areas. Among individual measures, LLITN use was associated with more malaria cases in urban areas (IRR 1.31, 95% CI 1.05–1.64, *p* = 0.018) and fewer cases in rural areas (IRR 0.74, 95% CI 0.55–0.98, *p* = 0.035). Use of insecticide sprays was associated with fewer cases in the full sample (IRR 0.85, 95% CI 0.70–1.02, *p* = 0.075). Mosquito coils and house modifications were not associated with malaria incidence in any analysis (Table 3).

**Table 3:** Negative binomial regression results for malaria preventive measures, community-based environmental management practices and malaria cases in the past year (*n* = 282).

Variable	Sample	IRR	95% CI	*p*-value
Use of malaria preventive measures	Full sample	0.624	0.33–1.18	0.147
	Rural area	0.397	0.17–0.92	0.030^**^
	Urban area	1.227	0.49–3.05	0.660
LLITNs	Full sample	1.075	0.89–1.29	0.439
	Rural area	0.735	0.55–0.98	0.035^**^
	Urban area	1.310	1.05–1.64	0.018^**^
Insecticide sprays	Full sample	0.845	0.70–1.02	0.075^*^
	Rural area	0.971	0.72–1.31	0.845
	Urban area	0.855	0.68–1.07	0.178
Mosquito coils	Full sample	1.085	0.91–1.30	0.376
	Rural area	1.130	0.86–1.49	0.388
	Urban area	1.022	0.82–1.28	0.848
House modifications	Full sample	1.106	0.84–1.46	0.475
	Rural area	0.940	0.63–1.39	0.759
	Urban area	1.192	0.84–1.70	0.329
Engagement in household-level environmental management practices	Full sample	0.921	0.50–1.71	0.793
	Rural area	0.528	0.24–1.18	0.119
	Urban area	2.563	0.94–6.96	0.065^*^
Eliminating standing water	Full sample	1.014	0.86–1.20	0.871
	Rural area	0.840	0.65–1.09	0.186
	Urban area	1.142	0.93–1.40	0.202
Managing trash cans	Full sample	0.863	0.74–1.01	0.071^*^
	Rural area	0.859	0.67–1.11	0.237
	Urban area	0.893	0.73–1.09	0.258
Mowing grass around the house	Full sample	1.048	0.88–1.25	0.607
	Rural area	0.905	0.69–1.18	0.468
	Urban area	1.210	0.97–1.51	0.096^*^
Maintaining drainage systems	Full sample	0.956	0.81–1.13	0.590
	Rural area	0.879	0.69–1.12	0.301
	Urban area	1.049	0.85–1.29	0.645
Covering water storage containers	Full sample	1.015	0.86–1.19	0.858
	Rural area	1.005	0.78–1.29	0.972
	Urban area	1.053	0.86–1.28	0.607
Sweeping the yard	Full sample	0.877	0.74–1.04	0.142
	Rural area	0.949	0.73–1.24	0.696
	Urban area	0.872	0.70–1.08	0.219
Other household practices	Full sample	1.234	1.05–1.45	0.010^**^
	Rural area	1.174	0.92–1.50	0.195
	Urban area	1.229	1.01–1.50	0.044^**^
Engagement in community-level environmental management practices	Full sample	1.233	0.89–1.70	0.203
	Rural area	0.663	0.40–1.11	0.119
	Urban area	1.646	1.11–2.44	0.014^**^
Removing stagnant water in open places (markets, hospitals)	Full sample	0.928	0.78–1.11	0.403
	Rural area	0.832	0.63–1.09	0.188
	Urban area	0.973	0.79–1.21	0.805
Mowing grass in open places	Full sample	1.028	0.87–1.21	0.740
	Rural area	0.801	0.63–1.02	0.075^*^
	Urban area	1.205	0.98–1.48	0.073^*^
Filling potholes	Full sample	1.052	0.88–1.26	0.589
	Rural area	0.815	0.61–1.08	0.159
	Urban area	1.185	0.95–1.48	0.132
Sweeping around markets	Full sample	0.940	0.80–1.10	0.436
	Rural area	0.768	0.60–0.98	0.036^**^
	Urban area	1.014	0.84–1.23	0.886
Information sharing	Full sample	0.987	0.83–1.17	0.879
	Rural area	0.749	0.57–0.98	0.034^**^
	Urban area	1.116	0.91–1.37	0.293

IRR, incidence rate ratio, CI, confidence interval. ^**^p < 0.05, ^*^p < 0.10.

Engagement in any household-level environmental management practice was associated with more malaria cases in urban areas at the 10% level (IRR 2.56, 95% CI 0.94–6.96, *p* = 0.065). Management of trash cans was associated with fewer cases in the full sample (IRR 0.86, 95% CI 0.74–1.01, *p* = 0.071) and mowing grass around the house with more cases in urban areas (IRR 1.21, 95% CI 0.97–1.51, *p* = 0.096), both at the 10% level. Reported frequency of other household environmental practices not listed in the survey was associated with more malaria cases in the full sample (IRR 1.23, 95% CI 1.05–1.45, *p* = 0.010) and in urban areas (IRR 1.23, 95% CI 1.01–1.50, *p* = 0.044) (Table 3).

Community engagement in the control of mosquito breeding sites was associated with more malaria cases in urban areas (IRR 1.65, 95% CI 1.11–2.44, *p* = 0.014). The corresponding rural estimate did not reach significance (IRR 0.66, 95% CI 0.40–1.11, *p* = 0.119). Among individual community-level practices, sweeping around markets (IRR 0.77, 95% CI 0.60–0.98, *p* = 0.036) and information sharing (IRR 0.75, 95% CI 0.57–0.98, *p* = 0.034) were associated with fewer malaria cases in rural areas. Mowing grass in open places was associated with more cases in urban areas and fewer cases in rural areas at the 10% level (urban IRR 1.20, *p* = 0.073; rural IRR 0.80, *p* = 0.075) (Table 3).

### 3.4 Barriers to sustaining environmental practices

Respondents identified factors they considered necessary to sustain engagement in environmental management practices within their communities. Continuous community education on malaria prevention and environmental management was the most frequently cited.

Among respondents who answered this question (*n* = 200), 56.5% reported active involvement in community activities aimed at eliminating mosquito breeding sites, 38.5% were not currently participating but expressed willingness to join, and 5% reported no interest.

Sustaining these practices was often limited by lack of resources. Respondents emphasized the need for equipment, financial support, and regular government-led cleanliness campaigns. Incentives such as small rewards or public recognition were also viewed as potential motivators for wider community participation. However, 50.4% of respondents reported challenges in maintaining environmental practices, mainly financial constraints (61.3%) and time limitations (49.3%).

The donut chart summarizes reported challenges to sustaining environmental practices, stratified by area of residence. The outer ring represents the proportion of respondents in urban (50.2%) and rural (50.7%) settings who reported experiencing at least one challenge. The inner ring shows the distribution of types of challenges conditional on area, separating financial constraints and time constraints within each group. Among urban respondents facing challenges, 54.8% cited financial constraints and 52.9% cited time constraints. In contrast, among rural respondents, 79.0% reported financial constraints and 39.5% reported time constraints. Percentages in the inner ring are calculated relative to respondents within the same area who reported challenges, rather than the total sample (Figure 1).

**Figure 1:**
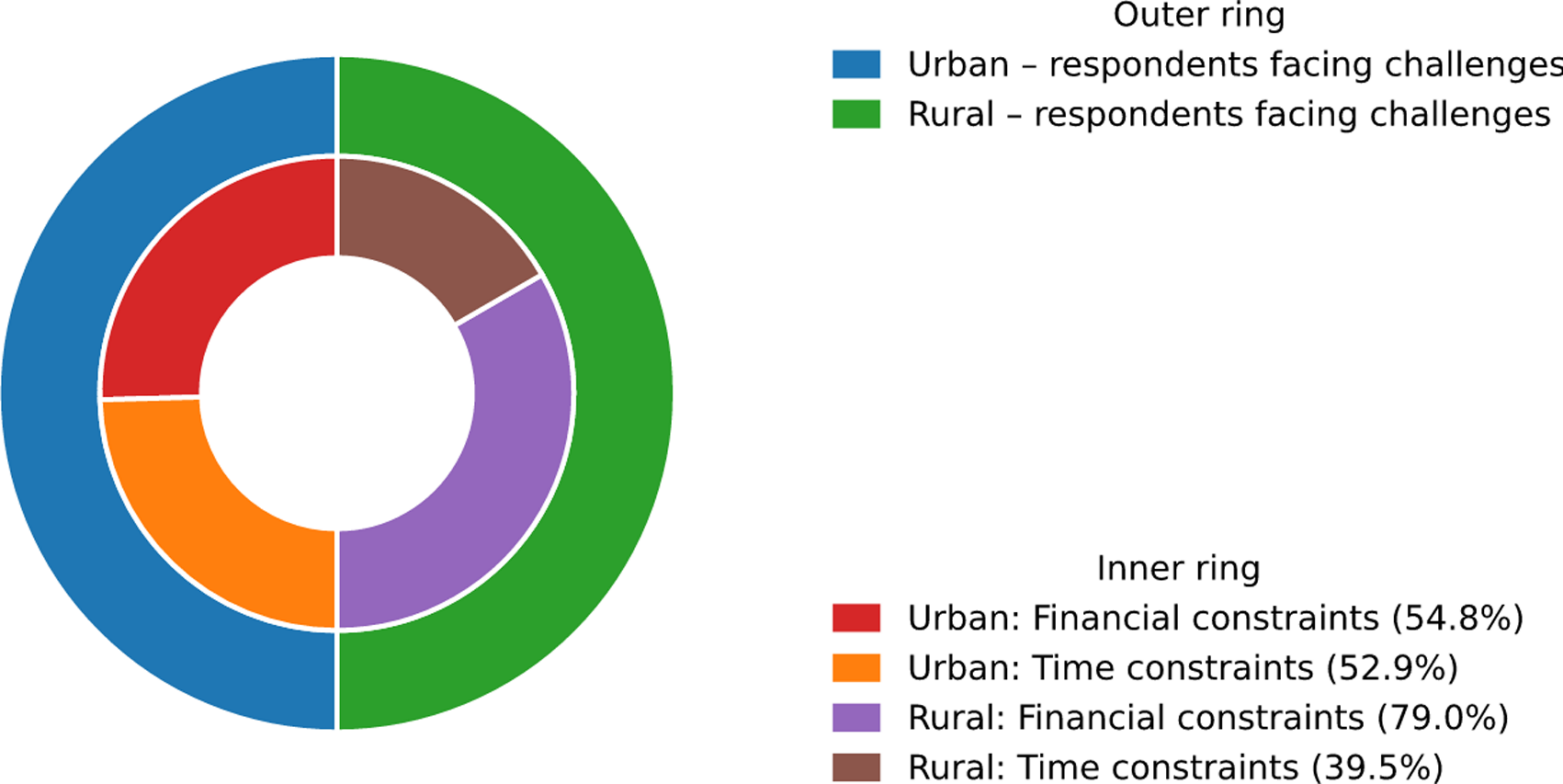
Challenges in sustaining environmental practices across urban and rural settings.

## 4 Discussion

In this study, the overall self-reported malaria incidence among participants was 62.1%, suggesting that malaria remains a major public health issue in Malawi. Although this level of self-reported infection conforms with findings from previous community-based surveys conducted in Sub-Saharan Africa, it shows persistent transmission despite widespread use of preventive interventions. The observed proportion was higher than that reported in similar studies in urban Kenya (28.1%) and Ghana (18.1%), but lower than rural surveys in DR Congo (96%) and Mozambique (66%) (21–24). The variations could be attributed to differences in study design, geographical setting, sample characteristics, and environmental conditions that differ between settings.

In the present study, the use of malaria preventive measures was high, with 95.4% of respondents reporting at least one method, mainly LLITNs. This finding is consistent with national malaria reports and studies in other African settings where LLITNs coverage has improved following mass distribution campaigns (25, 26). However, the higher usage did not correspond with a significant reduction in malaria incidence in the full sample. Because exposures were reported at the time of the survey while malaria cases were reported for the preceding 12 months, the possibility that net use followed rather than preceded malaria episodes cannot be excluded. Challenges related to correct or consistent use, net quality, or insecticide resistance may also contribute to the problem. A similar observation was made in Nigeria and Uganda, where owning LLITNs did not always mean reduced malaria burden (27, 28).

When stratified by area of residence, LLITN use was associated with a higher number of malaria cases in urban areas and a lower number in rural areas. This divergence should be interpreted with caution. A comparable absence of protection has been reported in Malawi at a national scale in an analysis of dried blood spots collected during the 2015–2016 Malawi Demographic and Health Survey, in which infection was confirmed by quantitative PCR. Neither living in a household owning a bed net, nor sleeping under an LLITN the previous night, was associated with a lower prevalence of *P. falciparum* infection among adolescents and adults, whereas urban residence, greater household wealth, and higher educational attainment were protective (29). In the present study, exposures were reported at the time of the survey, while malaria cases were reported for the preceding 12 months; the possibility that net use followed rather than preceded malaria episodes cannot be excluded. Residents of districts with higher transmission intensity may also be both more likely to use nets and more likely to experience malaria; however, district-level data were not collected in this study, so this potential confounding could not be assessed. Other studies have argued that factors such as infrequent replacement of worn nets, inconsistent use, and outdoor evening exposure occurring before net use begins may also contribute (30, 31). The present data do not allow these explanations to be distinguished, and the finding should not be read as evidence that LLITN use increases malaria risk.

Household-level environmental management practices were reported by most of the study participants, most commonly sweeping the yard and mowing grass. Few of these practices showed a statistically robust association with malaria incidence. Engagement in any household practice was associated with a higher number of cases in urban areas in unadjusted analysis, but this association attenuated and was no longer significant after adjustment for participant characteristics and area of residence. Only management of trash cans was associated with fewer cases in the full sample in unadjusted analysis. Studies in Ethiopia and Burkina Faso have similarly reported limited household participation in environmental control, often linked to low community mobilization, lack of tools, and weak local leadership support (32–34). Further research is needed to examine how consistently and effectively these practices are implemented, which self-reported measures of frequency cannot capture.

Engagement in community-level control of mosquito breeding sites showed contrasting associations by area of residence. In rural areas, engagement was associated with fewer malaria cases. In urban areas, engagement was associated with more malaria cases in both unadjusted and adjusted analyses. Previous studies in Malawi and Zambia have reported benefits of community cleanup campaigns, environmental education, and participatory monitoring (16, 35). The positive association observed here in urban areas may reflect the same limitations described above: the cross-sectional design cannot establish whether engagement preceded or followed malaria episodes, and unmeasured differences in local transmission intensity, infrastructure and environmental conditions between urban settings may account for the association. It can also be suggested that in urban areas, where drainage and waste management infrastructure is generally better developed, the marginal contribution of additional community breeding-site control may be smaller than in rural settings. These interpretations remain speculative and require confirmation in studies with longitudinal designs and geographically resolved data.

Despite promising participation levels, sustainability remains an issue. The most frequently reported barriers were financial constraints (61.3%) and time limitations (49.3%). These findings are consistent with those of community-based interventions in Kenya and Tanzania, where inadequate funding, lack of incentives, and absence of government-led maintenance programs hindered long-term engagement (13, 36). Respondents in this study also highlighted the importance of continuous education, material support, and recognition-based incentives to sustain community participation.

This study has several limitations. Participants were recruited by convenience sampling through social media platforms, and the survey was administered online. This approach necessarily excluded individuals without internet access or social media use, who constitute a substantial proportion of the Malawian population and are concentrated in rural areas. The resulting sample was accordingly unrepresentative of the national population in several respects: 73.4% of respondents resided in urban areas, compared with approximately 82% of the Malawian population living in rural areas; 92.6% reported higher education; and respondents were relatively young (mean age 29.3 years, range 18–52). The findings should therefore not be generalized to the wider Malawian population, and particularly not to rural communities, where the malaria burden is highest. A further consequence is that the level of education could not be examined as a covariate, since it was close to uniform across respondents. The questionnaire recorded area of residence only as a binary urban or rural classification, and no district-level or other geographic identifier was collected; the survey was kept deliberately short because participants were not compensated for their time, and additional location questions were judged to pose a risk to the anonymity of respondents. The survey was distributed through online communities whose members were drawn from different parts of Malawi, so respondents were not confined to a single locality, but their specific districts could not be determined. Another limitation was self-reported malaria incidence not verified by medical records, which may have introduced recall bias or misclassification. Febrile illnesses of other aetiologies are frequently attributed to malaria in endemic settings, and self-reported malaria has been shown to correspond imperfectly with parasitologically confirmed infection (18). The direction of any resulting misclassification cannot be determined from these data. The outcome was also recorded as the number of cases occurring within the respondent’s household rather than in the respondent alone (although household size was included as a covariate in the adjusted models reported in Supplementary Table S1). The cross-sectional study design limits causal inference and cannot establish the temporal relationship between malaria exposure and outcomes. Preventive measures reported may reflect responses to malaria rather than proactive practices. Positive associations found between malaria incidence and LLITN use or community engagement in urban areas should not be seen as indicative of increased risk. While adjustments for demographic factors did not significantly change estimates, the lack of district-level data is a limitation. Malaria transmission varies greatly across Malawi, so residents in high-transmission areas may adopt preventive measures while also experiencing more malaria cases. This residual confounding could explain the contradictory findings related to LLITN and community engagement between urban and rural contexts. The overall sample size was modest, and the rural stratum comprised only 75 respondents, which limits statistical power and reduces the stability of stratified and covariate-adjusted estimates. All reported associations should be regarded as exploratory rather than as established effects.

This study found that preventive measures and environmental management practices are both widely reported in this sample but that respondents face substantial difficulty in sustaining environmental practices, with financial and time constraints the most commonly cited barriers. Associations between these practices and self-reported malaria incidence were generally weak and differed in direction between urban and rural respondents and should be regarded as exploratory. To sustain and later scale these interventions, there is a need for regular community education, financial and logistical support, and incorporation of environmental management into existing malaria control programs. Strengthening partnerships between local authorities, health institutions, and community groups could enhance the long-term sustainability of malaria prevention initiatives across Malawi’s diverse epidemiological environment.

## Data Availability

The datasets presented in this study can be found in online repositories. The names of the repository/repositories and accession number(s) can be found at: https://github.com/Memory/Malaria_lab.
