# Supplementary material for: Household environmental management practices, community engagement, and self-reported malaria incidence in urban and rural communities of Malawi
Source: Front Public Health. 2026 Oct 14;14:1919768. doi: 10.3389/fpubh.2026.1919768 (PMC13713877; doi:10.3389/fpubh.2026.1919768)
Supplement: Supplementary file 1 [file Data_Sheet_1.DOCX]

**Household environmental management practices, community engagement, and self-reported malaria incidence in urban and rural communities of Malawi**

**Memory Magdalene Lungu^1^, Arsen Davitadze^1*^**

^1^ Institute of International Development and Partnership, ITMO University, St. Petersburg, Russia

* Correspondence:  
Arsen Davitadze 
davitadzeap@gmail.com

**Supplementary material**

Evaluating the role of household environmental management practices and community engagement in malaria prevention in Malawi

## **Table S1. Negative binomial regression of malaria cases in the past year, unadjusted and adjusted for area of residence, and stratified by area (n = 282)**

| Predictor | Full sample, unadjusted | | Full sample, adjusted for area | | Urban (n = 207) | | Rural (n = 75) | |
| --- | --- | --- | --- | --- | --- | --- | --- | --- |
|  | IRR (95% CI) | p | IRR (95% CI) | p | IRR (95% CI) | p | IRR (95% CI) | p |
| **Malaria preventive measures** | | | | | | | | |
| Any preventive measure (yes vs no) | 0.62 (0.33–1.18) | 0.147 | 0.72 (0.39–1.33) | 0.296 | 1.23 (0.49–3.05) | 0.660 | 0.40 (0.17–0.92) | 0.030 |
| LLITNs | 1.08 (0.89–1.29) | 0.439 | 1.09 (0.91–1.30) | 0.336 | 1.31 (1.05–1.64) | 0.018 | 0.74 (0.55–0.98) | 0.035 |
| Insecticide sprays | 0.85 (0.70–1.02) | 0.075 | 0.89 (0.74–1.07) | 0.202 | 0.85 (0.68–1.07) | 0.178 | 0.97 (0.72–1.31) | 0.845 |
| Mosquito coils | 1.08 (0.91–1.30) | 0.376 | 1.06 (0.89–1.26) | 0.541 | 1.02 (0.82–1.28) | 0.848 | 1.13 (0.86–1.49) | 0.388 |
| House modification | 1.11 (0.84–1.46) | 0.475 | 1.09 (0.84–1.43) | 0.510 | 1.19 (0.84–1.70) | 0.329 | 0.94 (0.63–1.39) | 0.759 |
| **Household-level environmental management practices** | | | | | | | | |
| Any household practice (yes vs no) | 0.92 (0.50–1.71) | 0.793 | 1.09 (0.60–2.00) | 0.769 | 2.56 (0.94–6.96) | 0.065 | 0.53 (0.24–1.18) | 0.119 |
| Eliminating standing water | 1.01 (0.86–1.20) | 0.871 | 1.04 (0.88–1.22) | 0.659 | 1.14 (0.93–1.40) | 0.202 | 0.84 (0.65–1.09) | 0.186 |
| Maintaining drainage systems | 0.96 (0.81–1.13) | 0.590 | 0.99 (0.84–1.16) | 0.880 | 1.05 (0.85–1.29) | 0.645 | 0.88 (0.69–1.12) | 0.301 |
| Mowing grass around the house | 1.05 (0.88–1.25) | 0.607 | 1.09 (0.92–1.30) | 0.309 | 1.21 (0.97–1.51) | 0.096 | 0.91 (0.69–1.18) | 0.468 |
| Covering water storage containers | 1.01 (0.86–1.19) | 0.858 | 1.04 (0.89–1.21) | 0.643 | 1.05 (0.86–1.28) | 0.607 | 1.00 (0.78–1.29) | 0.972 |
| Managing trash cans | 0.86 (0.74–1.01) | 0.071 | 0.88 (0.76–1.03) | 0.112 | 0.89 (0.73–1.09) | 0.258 | 0.86 (0.67–1.11) | 0.237 |
| Sweeping the yard | 0.88 (0.74–1.04) | 0.142 | 0.90 (0.76–1.06) | 0.207 | 0.87 (0.70–1.08) | 0.219 | 0.95 (0.73–1.24) | 0.696 |
| Other household practices | 1.23 (1.05–1.45) | 0.010 | 1.21 (1.04–1.41) | 0.016 | 1.23 (1.01–1.50) | 0.044 | 1.17 (0.92–1.50) | 0.195 |
| **Community-based environmental management practices** | | | | | | | | |
| Community engagement (yes vs no) | 1.23 (0.89–1.70) | 0.203 | 1.24 (0.90–1.69) | 0.185 | 1.65 (1.11–2.44) | 0.014 | 0.66 (0.40–1.11) | 0.119 |
| Removing stagnant water in open places | 0.93 (0.78–1.11) | 0.403 | 0.93 (0.78–1.10) | 0.383 | 0.97 (0.79–1.21) | 0.805 | 0.83 (0.63–1.09) | 0.188 |
| Mowing grass in open places | 1.03 (0.87–1.21) | 0.740 | 1.05 (0.90–1.23) | 0.542 | 1.20 (0.98–1.48) | 0.073 | 0.80 (0.63–1.02) | 0.075 |
| Filling potholes | 1.05 (0.88–1.26) | 0.589 | 1.06 (0.89–1.26) | 0.536 | 1.19 (0.95–1.48) | 0.132 | 0.81 (0.61–1.08) | 0.159 |
| Sweeping around markets | 0.94 (0.80–1.10) | 0.436 | 0.93 (0.80–1.09) | 0.373 | 1.01 (0.84–1.23) | 0.886 | 0.77 (0.60–0.98) | 0.036 |
| Information sharing | 0.99 (0.83–1.17) | 0.879 | 0.99 (0.84–1.17) | 0.933 | 1.12 (0.91–1.37) | 0.293 | 0.75 (0.57–0.98) | 0.034 |

IRR = incidence rate ratio; CI = confidence interval. Counts of malaria cases in the past year were the outcome in all models. Each row is a separate model containing the listed predictor only (unadjusted and stratified columns) or the listed predictor plus area of residence (adjusted column). Binary predictors are coded yes = 1 vs no = 0; frequency-based predictors are coded on an ordinal scale (never = 0, rarely = 1, monthly/weekly/daily = 2), so IRRs express the change in expected malaria cases per one-category increase in reported frequency.

Area of residence was itself strongly associated with malaria counts (urban vs rural IRR = 0.53, 95% CI 0.39–0.71, p < 0.001) but was not appreciably associated with most exposures, and adjustment did not materially alter any effect estimate or reverse the direction of any association.

Community-based practices were modelled over the full sample; respondents not engaged in community activities are coded as "never" for these items.

## **Table S2. Sensitivity analysis: identical models using untruncated malaria case counts (n = 282)**

| **Predictor** | **Full sample, unadjusted** | | **Full sample, adjusted for area** | | **Urban (n = 207)** | | **Rural (n = 75)** | |
| --- | --- | --- | --- | --- | --- | --- | --- | --- |
|  | **IRR (95% CI)** | **p** | **IRR (95% CI)** | **p** | **IRR (95% CI)** | **p** | **IRR (95% CI)** | **p** |
| **Malaria preventive measures** | | | | | | | | |
| Any preventive measure (yes vs no) | 0.65 (0.33–1.27) | 0.206 | 0.75 (0.39–1.45) | 0.396 | 1.27 (0.49–3.28) | 0.618 | 0.42 (0.17–1.02) | 0.056 |
| LLITNs | 1.11 (0.92–1.34) | 0.274 | 1.13 (0.94–1.36) | 0.191 | 1.35 (1.07–1.70) | 0.012 | 0.77 (0.57–1.04) | 0.085 |
| Insecticide sprays | 0.83 (0.68–1.00) | 0.052 | 0.87 (0.72–1.04) | 0.130 | 0.82 (0.65–1.04) | 0.095 | 0.98 (0.72–1.34) | 0.915 |
| Mosquito coils | 1.15 (0.96–1.38) | 0.138 | 1.12 (0.94–1.34) | 0.218 | 1.08 (0.86–1.37) | 0.487 | 1.20 (0.90–1.59) | 0.216 |
| House modification | 1.08 (0.81–1.44) | 0.612 | 1.07 (0.81–1.41) | 0.648 | 1.16 (0.80–1.68) | 0.425 | 0.91 (0.61–1.38) | 0.672 |
| **Household-level environmental management practices** | | | | | | | | |
| Any household practice (yes vs no) | 0.96 (0.50–1.82) | 0.896 | 1.15 (0.61–2.16) | 0.663 | 2.66 (0.95–7.41) | 0.062 | 0.55 (0.24–1.30) | 0.173 |
| Eliminating standing water | 1.00 (0.84–1.18) | 0.959 | 1.02 (0.86–1.21) | 0.797 | 1.13 (0.92–1.40) | 0.253 | 0.81 (0.62–1.07) | 0.135 |
| Maintaining drainage systems | 0.96 (0.81–1.14) | 0.654 | 1.00 (0.85–1.18) | 0.966 | 1.09 (0.89–1.35) | 0.404 | 0.84 (0.66–1.09) | 0.189 |
| Mowing grass around the house | 1.06 (0.88–1.28) | 0.519 | 1.12 (0.93–1.34) | 0.234 | 1.24 (0.99–1.57) | 0.065 | 0.90 (0.68–1.20) | 0.484 |
| Covering water storage containers | 1.04 (0.88–1.22) | 0.670 | 1.06 (0.91–1.25) | 0.453 | 1.09 (0.89–1.34) | 0.408 | 1.01 (0.78–1.31) | 0.953 |
| Managing trash cans | 0.87 (0.74–1.03) | 0.106 | 0.90 (0.76–1.06) | 0.193 | 0.93 (0.76–1.14) | 0.497 | 0.83 (0.64–1.07) | 0.153 |
| Sweeping the yard | 0.86 (0.72–1.03) | 0.106 | 0.88 (0.74–1.05) | 0.151 | 0.85 (0.67–1.07) | 0.159 | 0.94 (0.72–1.24) | 0.668 |
| Other household practices | 1.30 (1.10–1.53) | 0.002 | 1.27 (1.08–1.49) | 0.003 | 1.29 (1.05–1.59) | 0.015 | 1.23 (0.96–1.58) | 0.105 |
| **Community-based environmental management practices** | | | | | | | | |
| Community engagement (yes vs no) | 1.30 (0.93–1.81) | 0.129 | 1.30 (0.94–1.81) | 0.111 | 1.72 (1.14–2.59) | 0.009 | 0.70 (0.41–1.21) | 0.204 |
| Removing stagnant water in open places | 0.92 (0.77–1.10) | 0.356 | 0.91 (0.77–1.09) | 0.301 | 0.93 (0.75–1.16) | 0.533 | 0.87 (0.65–1.15) | 0.324 |
| Mowing grass in open places | 1.06 (0.90–1.26) | 0.477 | 1.09 (0.92–1.29) | 0.302 | 1.25 (1.01–1.55) | 0.036 | 0.82 (0.64–1.06) | 0.136 |
| Filling potholes | 1.08 (0.90–1.31) | 0.408 | 1.10 (0.92–1.32) | 0.311 | 1.25 (1.00–1.57) | 0.050 | 0.80 (0.59–1.08) | 0.139 |
| Sweeping around markets | 0.97 (0.82–1.14) | 0.706 | 0.97 (0.83–1.13) | 0.666 | 1.05 (0.86–1.28) | 0.617 | 0.78 (0.60–1.02) | 0.066 |
| Information sharing | 1.01 (0.85–1.21) | 0.886 | 1.03 (0.87–1.22) | 0.750 | 1.17 (0.95–1.45) | 0.138 | 0.74 (0.56–0.97) | 0.031 |

Models in Table S1 apply the rule that reported case counts exceeding 10 were replaced by the reported household size, since a household cannot experience more episodes than it has members; this affected four observations (reported values 11, 12, 12 and 20). Table S2 repeats every model using the untruncated counts as reported by participants. Estimates are materially unchanged and no conclusion depends on this decision. Area of residence in the untruncated data: urban vs rural IRR = 0.52, 95% CI 0.38–0.71, p < 0.001.
